# The Haematological and Biochemical Responses of Goat Bucks to Range Grazing and Indoor Concentrate‐Based Feeding Systems

**DOI:** 10.1002/vms3.71221

**Published:** 2026-09-10

**Authors:** Selamu Abraham, Yisehak Kechero, Jane Wamatu

**Affiliations:** ^1^ Department of Animal Sciences Arba Minch University Arba Minch Ethiopia; ^2^ Department of Animal Science Wachemo University Hosaena Ethiopia; ^3^ International Center for Agricultural Research in the Dry Areas Addis Ababa Ethiopia

**Keywords:** biochemical profiles, breeding bucks, haematological parameters, serum electrolytes

## Abstract

**Background:**

Range grazing remains the principal feed resource for goats in smallholder production systems, particularly in regions where range grazing and intensive concentrate‐based systems coexist. However, expanding cropland has reduced the availability of grazing lands and shifted production toward concentrate‐based feeding systems.

**Objective:**

This study aimed to evaluate the haematological and biochemical responses of goat bucks managed under range grazing and indoor concentrate‐based feeding systems.

**Methods:**

Forty yearling bucks (10 bucks/treatment) were randomly allocated to one of four 90‐day feeding groups: range grazing (T1) or three treatments with roughage‐to‐concentrate ratios of: 60:40 (T2), 50:50 (T3) and 40:60 (T4) on a DM basis. Blood samples (10 mL/animal) were collected every fortnight via jugular vein from all bucks and were subjected to haematological and biochemical analysis.

**Results:**

Bucks in T3 and T4 had significantly (*p* < 0.05) greater haemoglobin, red blood cell and packed cell volume compared to T1; nevertheless, the difference between T1 and T2 was not significant. Serum protein, albumin, glucose and testosterone concentrations were also higher in bucks fed concentrate diets (*p* < 0.05). White blood cells, mean corpuscular volume, mean corpuscular haemoglobin concentration, the red blood cell distribution widths, globulin, aspartate aminotransferase, alanine aminotransferase, alkaline phosphatase, urea and creatinine remained unaffected (*p* > 0.05). Calcium level was significantly higher in T4 than T1 (*p* < 0.05), while K, Na, Cl and P remained unchanged (*p* > 0.05).

**Conclusion:**

Bucks kept indoors on concentrate‐based diets showed better oxygen‐carrying capacity, iron status, nitrogen balance and body protein reserves than bucks reared under range grazing.

## 1 Introduction

Goats have been a part of the evolution of human civilization tracing back to the Neolithic period (Askar et al. 2015), as they are the first domesticated ungulates (Hermes et al. 2020). The animals play a vital role in ensuring food security in areas where other agricultural activities are challenging. The United Nations proposed 17 Sustainable Development Goals, from no poverty and zero hunger to climate action. In achieving this plan, the role of goats is crucial, particularly relevant to food and nutritional sustainability, as well as economic and environmental sustainability (Lu 2023). However, these animals face escalating threats from climate‐induced stresses, including recurrent droughts, erratic rainfalls and heatwaves, which severely compromise the availability and nutritional quality of natural grazing pastures, thereby threatening the sustainability of range‐based goat production systems (Mugoti et al. 2025). Smallholder goat producers, particularly in tropical and subtropical countries, rely heavily on seasonal natural pastures and crop residues. While this system provides the least expensive source of nutrients, it exposes animals to periodic nutritional deficits, disease risks and thermal stress, especially during dry seasons (Soul et al. 2019). Under this production system, animals often fail to meet their nutritional requirements, and a deficit is further aggravated by seasonal forage fluctuations, anti‐nutritional plant compounds and endemic parasitism (Waghorn 2008; Cooke et al. 2025), leading to chronic undernutrition that constrains growth and health, especially during dry seasons (Cooke et al. 2025).

Concentrate feeding is a practical strategy to meet the nutrient requirements of range animals while improving growth and health (Karunanayaka et al. 2022). It also manipulates forage consumption and digestibility, decreases energy costs associated with grazing activity, and reduces the time spent on grazing (Mahgoub et al. 2025); as a result, the efficiency of fodder utilization is positively influenced (Askar et al. 2021). Moreover, concentrate diets actively manipulate rumen fermentation dynamics, reducing methane emissions per unit of product (Ma et al. 2025), and also minimizing overgrazing pressure on pastures, thereby promoting vegetation recovery and reducing land degradation (Askar et al. 2021). Feeding concentrates also improves overall productivity by supplying limiting nutrients during critical feed shortages, thereby reducing morbidity and mortality (Mellado et al. 2020). In this regard, Sadrarhami et al. (2022) reported higher daily weight gain and loin thickness in lambs fed concentrate‐based diets than those managed under nomadic grazing. Similarly, Barbari goats (Indian goat) fed a concentrate‐mixed diet under an intensive feeding system experienced improved growth, nutrient utilization and blood profile (Dutta et al. 2025).

The haematological and biochemical parameters of blood can be used to monitor and evaluate the health, nutritional and physiological status of animals. Changes in the number of white and red blood cells, the packed cell volume, haemoglobin concentration and enzymatic activity can show changes in the immune system, oxygen‐carrying capacity and overall physiological resilience (Arsenopoulos et al. 2025). Likewise, serum metabolites, total protein, albumin, urea, creatinine and electrolytes provide insight into energy and protein metabolism, organ function and the adequacy of dietary supply (Soul et al. 2019).

While numerous studies have reported the effects of various diets, forage species and agro‐industrial by‐products on the health profiles of goats under controlled conditions, there is a limitation of comparative studies examining how different production systems, particularly extensive range grazing versus indoor concentrate‐based management, influence the haematological and biochemical responses of goat bucks. In addition, most existing studies focus on growth performance, carcass yield and quality, lactating goats and growing kids, with limited attention on breeding bucks. This gap is particularly evident in tropical agro‐ecologies, where grazing goats face seasonal nutrient deficits, parasitic burdens and oxidative stress, potentially amplifying health vulnerabilities. Therefore, monitoring these biomarkers provides critical insights into how different feeding and production systems affect systemic health and wellness. To this end, the objective of this study was to evaluate the effect of range grazing and indoor concentrate‐based production systems on haematological and biochemical indices of goat bucks. The study hypothesized that a concentrate‐based feeding system would improve the systemic health and wellness of the goat bucks. These blood‐based indices, therefore, offer a robust tool to evaluate how different feeding and management systems influence the internal environment of goats beyond what can be inferred from performance traits alone.

## 2 Materials and Methods

### 2.1 The Study Area

Woyto‐Guji goat bucks (an indigenous breed of the Konso Zone, Ethiopia) were used for this study. The experimental animals were sourced from smallholder farmers participating in the community‐based breeding programme in villages such as Bayde, Arfayde and Jarso in the Konso Zone, southern Ethiopia. The study was conducted at the Bayde station, located in the Karat Zuria rural district of the Konso Zone in southern Ethiopia. The Konso Zone is located 535 km southwest of Addis Ababa, Ethiopia. It is situated between 501 and 2000 m above sea level at latitudes 50°10′0′′ to 50°40′0′′N and longitudes 37°00′0′′ to 37°45′0′′E. The zone receives a mean annual rainfall of about 800 mm, with an average annual temperature of 23.5°C (Kasho et al. 2022).

### 2.2 Experimental Treatments and Design

The experiment involved 40 intact yearling bucks with an average initial body weight of 22.34 ± 0.27 kg. Prior to the commencement of the study, all the animals were clinically examined by a veterinarian to confirm their health status and normal physiological condition. The examination included assessments of grazing ability, movement activity, limb, mouth and ruminal activity and body temperature. The animals were treated for ectoparasites with ivermectin (0.2 mg/kg) and for endoparasites with albendazole (7.5 mg/kg). Following a 15‐day adaptation period, the animals were divided into four different groups in a randomized complete block design (RCBD), with 4 × 4 arrangement (4 treatments and 4 blocks). The bucks were blocked according to their initial body weight: Block‐1 = 18–20 kg, Block‐2 = 21–22 kg, Block‐3 = 23–24 kg and Block‐4 = 25–26.5 kg. Each treatment consisted of a total of 10 bucks. The treatments were as follows:

- **T1**: Range grazing on selected grazing fields (control)
- **T2**: Indoor feeding at roughage‐to‐concentrate ratio of 60:40 on the DM basis
- **T3**: Indoor feeding at roughage‐to‐concentrate ratio of 50:50 on the DM basis
- **T4**: Indoor feeding at roughage‐to‐concentrate ratio of 40:60 on the DM basis.

**Range grazing (T1)**: Goat bucks were kept under outdoor grazing and obtained their feed from natural pasture with exposure to environmental conditions and normal physical activity.

**Indoor concentrate‐based feeding system (T2‒T4)**: Goat bucks were housed indoors and fed controlled diets composed of different roughage‐to‐concentrate ratios under a controlled environment.

The roughage‐to‐concentrate ratios were selected purposefully to represent a controlled gradient of concentrate supplementation commonly used in production and experimental studies. The ratios cover the full range from all‐forage (T1) through moderate (T2 and T3) to high concentrate (T4) feeding. This span reflects practical feeding strategies used across production stages (maintenance, growing and finishing) and enables extrapolation of responses across that production continuum. The proportion of forage was calculated on a dry‐matter basis from the weighed feed ingredients.

The range grazing animals (T1) were reared in four specific grazing fields (approximately 25 ha) in Bayde area of the Karat Zuria rural district, southern Ethiopia. The animals were rotated among these fields every 5 days during the 90‐day experimental period. The grazing fields were rented from smallholder farmers for 90 days and enclosed to keep out other animals. The stocking rate was 0.4 (10 bucks/25 ha), and grazing capacity was 2.5 (25 ha/10 bucks), which represents the reciprocal of grazing land available per animal over the 90‐day trial. Since both the flock size and total grazing land were fixed, the overall stocking rate remained constant. The grazing fields are rich in botanical diversity, featuring dominant native species such as *Cynodon dactylon, Becium grandiflorum*, sedges and forbs. Range grazing bucks were kept in a loose housing system and allowed to graze throughout the day (about 10 h per day) on the range pastures. Bucks allocated to concentrate‐based production systems (T2, T3 and T4) were managed indoors in a single pen (1.2 × 1.5 m) per animal. Sand, wood shavings and straw were used as bedding material and replaced every single day. The animals in these treatments were fed on different roughage‐to‐concentrate ratios. The roughage source feeds were the principal feeds available in grazing/browsing lands of the study zone. These included major foliage types (*Tremenalia brownie* and *Cordia africana*), natural pastures (*C. dactylon, B. grandiflorum*, sedges and forbs) and crop residues (maize and sorghum stover). The feeds were harvested locally, dried in a shed to achieve a moisture content of 15%, and then mixed in equal proportions.

The commercial concentrate, consisting of 33% maize grain, 40% wheat bran, 15% wheat middlings, 10% noug cake, 1% vitamin premix and 1% salt, was purchased from the Gamo Gofa Farmers’ Vegetable, Fruit and Livestock Feed Marketing Co‐operative Union, Arba Minch. The animals were fed at a rate of 3% of metabolic body weight. Feeds were given in two equal portions every day, at 8:00 AM and 6:00 PM. Refusals were collected, and their weight was recorded daily, and intake was calculated as offered minus refused. The mean dry matter intake (DMI) was 334 g/day for T2, 351 g/day for T3 and 380 g/day for T4; as the amount of feed not given to T1 bucks was measured, DMI was not calculated. The animals had free access to clean water throughout the study period (NRC 2007).

### 2.3 Laboratory Analysis of the Diets

The nutritional compositions of the treatment diets are presented in Table 1. The samples were subjected to laboratory analysis in triplicate for all traits measured in this study. The dry matter content was determined by drying the samples in an oven at 105°C for 16 h, and the ash content was determined through burning in a furnace at 550°C for 3 h. The CP content was determined indirectly using Kjeldahl procedures (acid digestion, steam distillation and titration) where the nitrogen content was multiplied by a factor of 6.25 (CP = *N* × 6.25). The Soxhlet extraction (a solid‒liquid extraction) technique was used to estimate the ether extract content through a stepwise process: evaporation, condensation, immersion extraction, siphoning and recycling, with the solvent returned to the flask (AOAC 2019). The neutral detergent fibre (NDF) content of the samples was determined by pre‐treating the samples with heat‐stable amylase (Van Soest et al. 1991), followed by acid detergent fibre (ADF) and acid detergent lignin (ADL) (Van Soest and Robertson 1985). The metabolizable energy (ME) and total digestible nutrients (TDN) were assessed using the formulas given by Weiss et al. (1992). The percentage of nitrogen‐free extract (NFE) was estimated via the following formula:

(1) NFE%=100−(CP%+EE%+CF%+Ash%)

(2) TDN%=(0.90×%NFE)+(0.75×%CP)+(2.25×0.90×%EE)

(3) ME(MJ/kg)=TDN×0.15104

where ME = metabolizable energy, TDN = total digestible nutrients, NFE = nitrogen‐free extract, CF = crude fibre and CP = crude protein.

**TABLE 1:** Roughage to concentrate ratios and the nutritional composition of the treatment diets (DM basis, (%)).

	Treatments			
	T1	T2	T3	T4
Roughage (RF)	100%	60%	50%	40%
Concentrate (CC)	0%	40%	50%	60%
DM	87.47	88.80	92.32	90.80
Ash	10.07	12.47	12.85	13.76
CP	7.05	10.21	12.70	15.00
EE	0.98	1.34	2.04	2.82
CF	17	12.67	11.05	10.73
NFE	64.90	63.31	61.36	57.69
NDF	58.82	40.60	32.72	30.41
ADF	30.22	27.72	25.41	22.86
ADL	8.11	9.94	8.59	8.54
TDN	64.90	63.31	61.36	57.69
ME (MJ/kg)	11.20	11.31	11.35	11.33

*Note*: T1 denotes nutritional compositions of range fodders available for grazing goats.

Abbreviations: ADF, acid detergent fibre; ADL, acid detergent lignin; CC, commercial concentrate; CP, crude protein; DM, dry matter; EE, ether extract; ME, metabolizable energy; NDF, neutral detergent fibre; NFE, nitrogen‐free extract; RF, range fodders; TDN, total digestible nutrients.

### 2.4 Blood Sampling and Haemato‐Biochemical Analysis

Following a 15‐day adaptation period, blood samples were collected from all bucks (*n* = 10 per treatment) at 15‐day intervals throughout the 90‐day experimental period, resulting in six samples per animal. Approximately 10 mL of blood was drawn via jugular venepuncture using plastic disposable syringes before the morning meal, between 6:00 and 7:30 AM. About 5 mL was used for haematological analysis, and the other 5 mL was used for serum chemistry. For the haematological analysis, the blood samples were transferred into sterile test tubes containing ethylenediaminetetraacetic acid (EDTA) and sent to the laboratory of Nech‐Sar Hospital, Arba Minch, Ethiopia. Blood samples deprived of anticoagulant were allowed to coagulate, centrifuged at 3000 rpm at 21°C for 10 min, and stored at −20°C for biochemical analysis (Roy et al. 2024). Haematological parameters, including RBC, WBC, PCV, Hb, MCV, MCH, MCHC, RDW‐CV and RDW‐SD, were analysed via a Sysmex XT 2000i analyser (VLD‐DPM‐CBC‐06, Mundelein, IL 60060, USA). The serum metabolites, including total protein (Biuret method), albumin (bromocresol green method), globulin (total protein minus albumin), glucose, testosterone and serum enzymes including aspartate aminotransferase (AST), alanine aminotransferase (ALT), alkaline phosphatase (AP), urea and creatinine, were determined using commercially available kits according to the manufacturer's instructions (Cogent of Span Diagnostic Ltd., Surat, Gujarat, India). Potassium, sodium and chloride were further investigated via the ion‐selective electrode (ISE) module of the Roche/Hitachi Cobas c systems (Model c501). Total calcium and phosphorus concentrations were determined using flame atomic absorption spectrophotometry and the colourimetry spectrophotometric method, respectively.

### 2.5 Statistical Analysis

Data were analysed using SAS‐JMP Pro 19 software by two‐way analysis of variance (ANOVA). Mean separation was performed using Tukey's HSD test, and differences were considered significant at *p* < 0.05 with a 95% confidence level and 80% (1−*β* = 0.80) statistical power. Blood samples were collected from 40 bucks (10 bucks per treatment) over six consecutive weeks. The following model was used for the analysis:

(4) Y(ijk)=μ+T(i)+β(j)+εijk

where *Y* = the response variables (haematological and plasma biochemical traits), *μ* = overall mean; *T*i = the fixed effect of dietary treatments (1, 2, 3, 4), *β*j = the block effect (*β*1 = 18–20 kg, *β*2 = 21–22 kg, *β*3 = 23–24 kg and *β*4 = 25–26.5 kg) and ε (ijk) = the random error. The Pearson correlation coefficient was used to determine the degree of association among haematological parameters as well as serum biochemical indicators. The strength and significance of these linear associations were determined via the correlation coefficient (*r*), with significance set at *p* < 0.05, *p* < 0.01 and/or *p* < 0.0001.

## 3 Results

### 3.1 Haematological Parameters

The haematological profile of the animals is presented in Table 2. The treatments had a reasonable influence (*p* < 0.05) on the Hb concentration, RBC count and PCV values. Bucks in T3 and T4 exhibited significantly (*p* < 0.05) greater Hb, RBC and PCV levels than those in T1; nevertheless, the difference was not statistically significant compared with that in T2. In contrast, the mean MCV, MCH, MCHC, WBC and red cell distribution widths (RDW‐CV and RDW‐SD) did not vary among the treatment groups (*p* > 0.05).

**TABLE 2:** Changes in haematological attributes of bucks managed under range grazing and concentrate‐based production systems.

	Dietary treatments					
Traits	T1	T2	T3	T4	SEM	*p*‐value
Hb (g/dL)	8.16^b^	8.82^ab^	9.87^a^	9.78^a^	0.27	0.0048
RBC (×10^6^/µL)	8.52^c^	10.29^bc^	12.45^a^	11.60^ab^	0.43	0.0006
PCV (%)	22.98^c^	26.93^bc^	35.97^a^	29.90^ab^	1.53	0.0013
MCV (fL)	21	20.93	21.37	21.90	0.26	0.0963
MCH (pg)	6.19	6.42	6.30	7.03	0.24	0.1284
MCHC (g/dL)	30.78	32.26	31.29	32.65	0.64	0.2217
WBC (×10^3^)	14.14	13.65	14.42	16.16	1.53	0.6874
RDW‐CV (%)	11.70	11.87	12.27	11.90	0.56	0.9073
RDW‐SD (fL)	14.90	15.20	15.73	15.27	0.88	0.9254

*Note*: Levels connected by different letters are significantly different.

Abbreviations: Hb, haemoglobin; MCH, mean corpuscular haemoglobin; MCHC, mean corpuscular haemoglobin concentration; MCV, mean corpuscular volume; PCV, packed cell volume; RBC, red blood cell count; RDW‐CV, red cell distribution coefficient of variation; RDW‐SD, red cell distribution standard deviation; WBC, white blood cells.

### 3.2 Biochemical Indices

The biochemical profile of the goat bucks is presented in Table 3. Concentrate‐based diets resulted in considerably increased serum protein (*p* = 0.0014) and albumin (*p* = 0.0075) levels across the treatment groups. The glucose concentration also increased in the concentrate‐fed bucks compared with the range grazing group (*p* < 0.05). Similarly, bucks in a concentrate‐based production system exhibited considerably greater testosterone concentrations than those reared under range grazing. However, serum globulin, albumin‐to‐globulin ratio, AST, ALT, AP, urea and creatinine were not affected.

**TABLE 3:** Biochemical attributes of bucks from different production systems.

	Dietary treatments					
Traits	T1	T2	T3	T4	SEM	*p*‐value
Total protein (g/dL)	6.22^b^	7.67^a^	8.95^a^	7.86^a^	0.37	0.0014
Albumin (g/dL)	2.51^b^	3.52^a^	3.55^a^	3.87^a^	0.21	0.0075
Globulin (g/dL)	3.71	4.31	5.08	4.14	0.36	0.1263
ALB/GLU (g/dL)	0.67	0.82	0.70	0.94	0.11	0.6712
Glucose (mg/dL)	43.50^b^	51.23^a^	56.25^a^	53.75^a^	1.52	0.0012
Testosterone (ng/mL)	8.50^b^	12.62^a^	16.25^a^	13.30^a^	0.90	0.0014
AST (μ/L)	78.68	74.57	73.21	71.50	1.59	0.0565
ALT (μ/L)	22.01	20.68	19.50	21.56	0.68	0.1137
AP (μ/L)	175.06	181.68	180.57	184.83	3.36	0.2861
Urea (mmol/L)	6.29	5.91	6.53	6.19	0.44	0.7966
Creatinine (mg/dL)	0.83	0.82	0.87	0.88	0.07	0.8988

*Note*: Levels not connected by the same letter in rows are significantly different.

Abbreviations: ALB/GLU, Albumin‐to‐glucose ratio; ALT, alanine aminotransferase; AP, alkaline phosphatase; AST, aspartate aminotransferase.

### 3.3 Serum Electrolytes

The serum mineral compositions for the experimental animals are presented in Table 4. The potassium (K), sodium (Na), chloride (Cl) and phosphorus (P) concentrations ranged from 4.58 to 4.86 mmol/L, 148.93 to 151.87 mmol/L, 112.77 to 115.07 mmol/L and 1.65 to 1.88 mmol/L, respectively, which did not significantly vary among the production systems (*p* > 0.05). On the other hand, the production systems had a meaningful effect (*p* < 0.05) on the calcium level. Bucks in T1 presented lower concentrations (2.19 mmol/L) than those in T4 (2.80 mmol/L), whereas significant variation was not detected among T1, T2 and T3.

**TABLE 4:** Serum electrolytes of bucks from range grazing and concentrate‐based production systems (mmol/L).

	Dietary treatments					
Traits	T1	T2	T3	T4	SEM	*p*‐value
K (mmol/L)	4.58	4.86	4.85	4.60	0.15	0.4385
Na (mmol/L)	151.87	149.77	148.93	149.57	0.89	0.1809
Cl (mmol/L)	115.07	114.53	115.40	112.77	0.79	0.1580
Ca (mmol/L)	2.19^b^	2.68^ab^	2.72^ab^	2.80^a^	0.09	0.0014
P (mmol/L)	1.65	1.84	1.72	1.88	0.18	0.7820

*Note*: Levels connected by different letters in rows are significantly different.

Abbreviation: SEM, standard error of the mean.

### 3.4 Correlations Among Haematological Parameters

The correlation matrix among the haematological profiles is presented in Table 5. RBCs were significantly positively correlated with Hb (*r* = 0.71, *p* < 0.01) and PCV (*r* = 0.67, *p* < 0.05), suggesting that Hb and PCV changes closely reflect RBC variations. The MCH also exhibited a strong positive correlation with the MCHC (*r* = 0.90, *p* < 0.0001). WBC was weakly associated with other haematological parameters (e.g., RBC [*r* = 0.02, *p* > 0.05], Hb [*r* = 0.21, *p* > 0.05], MCV [r = 0.40, *p* > 0.05] and MCH [*r* = ‐0.13, *p* > 0.05]). RDW‐CV and RDW‐SD were nearly perfectly correlated with each other (*r* = 0.99, *p* < 0.0001). On the other hand, both RDW‐CV and RDW‐SD were strongly negatively correlated with MCH (*r* = −0.52, *p* < 0.01) and MCHC (*r* = −0.51, *p* < 0.01).

**TABLE 5:** Correlation matrix of the haematological parameters.

Traits	Hb	PCV	RBC	MCV	MCH	MCHC	RDW‐CV	RDW‐SD	WBC
Hb	1	0.80***	0.71**	0.30	0.33	−0.17	0.10	0.11	0.05
PCV		1	0.67*	0.44	0.22	−0.0.47	0.01	0.004	0.28
RBC			1	0.54*	0.21	−0.0.15	0.32	0.31	0.21
MCV				1	0.35	−0.0.08	0.11	0.091	0.40
MCH					1	0.90***	−0.0.52*	−0.0.52*	−0.0.13
MCHC						1	−0.0.51*	−0.0.51*	−0.0.04
RDW‐CV							1	0.99***	0.03
RDW‐SD								1	0.006
WBC									1

Abbreviations: Hb, haemoglobin; MCH, mean corpuscular haemoglobin; MCHC, mean corpuscular haemoglobin concentration; MCV, mean corpuscular volume; PCV, packed cell volume; RBC, red blood cell; RDW‐CV, red cell distribution coefficient of variation; RDW‐SD, red cell distribution standard deviation; WBC, white blood cell.

*p < 0.05, **p < 0.01 and ***p < 0.0001 show the strength and significance of association between traits.

### 3.5 Correlations Among Serum Biochemical Indices

The results of the Pearson correlation analysis of the serological biomarkers are presented in Table 6. The serum protein concentration was strongly correlated with the albumin (*r* = 0.71, *p* < 0.01), testosterone (*r* = 0.72, *p* < 0.01) and glucose concentrations (*r* = 0.53, *p* < 0.05). Albumin also exhibited a significant positive correlation with the albumin‐to‐globulin ratio (*r* = 0.56, *p* < 0.05) and testosterone level (*r* = 0.74, *p* < 0.01). The albumin‐globulin ratio was strongly negatively correlated with globulin (*r* = −0.64, *p* < 0.05), which is predictable since this ratio is inversely related to the globulin level. Testosterone was significantly positively correlated with glucose concentration (*r* = 0.64, *p* < 0.05). AP also exhibited a strong positive correlation with glucose concentrations *(r* = 0.53, *p* < 0.05). The correlation among ALT, AST, AP, urea and creatinine and with other biochemical parameters was weak (*p* > 0.05).

**TABLE 6:** Pearson's correlation matrix among the serological parameters.

Traits	Protein	Albumin	Globulin	ALB/GLB	Glucose	Testosterone	AST	ALT	AP	Urea	Creatinine
Protein	1	0.71**	0.85***	−0.0.16	0.53*	0.72**	0.01	0.42	0.36	−0.0.06	−0.0.16
Albumin		1	0.24	0.56*	0.44	0.74**	0.15	0.43	0.33	0.1	−0.0.05
Globulin			1	−0.64	0.41	0.45	−0.0.09	0.26	0.25	−0.0.08	−0.0.18
ALB/GLB				1	0.03	0.24	0.24	0.12	0.02	0.12	0.14
Glucose					1	0.64*	−0.0.06	0.42	0.43	−0.0.15	−0.0.3
Testosterone						1	0.11	0.37	0.39	0.11	−0.0.21
AST							1	0.25	−0.0.24	0.3	−0.0.01
ALT								1	0.3	0.41	−0.0.27
AP									1	0.12	0.03
Urea										1	0.12
Creatinine											1

Abbreviations: ALB/GLB, Albumin‐to‐globulin ratio; ALT, alanine aminotransferase; AP, alkaline phosphatase; AST, aspartate aminotransferase.

*p < 0.05, **p < 0.01 and ***p < 0.0001 show the strength and significance of association between traits.

## 4 Discussion

### 4.1 Haematological Responses of the Animals

Haematological responses are widely reported; haematological variables are used to evaluate health conditions and physiological status in detail and to assess environmental stresses (Arsenopoulos et al. 2025). Furthermore, haematology analysis is a valuable tool for assessing the quality of feeds, such as nutrient availability, nutrient deficiencies and digestibility (Yusuf et al. 2021; Zaher et al. 2020). In the present study, Hb and RBC were greater in bucks fed T3 and T4 diets than those in T1, indicating relatively greater erythropoiesis and oxygen‐carrying capacity than in bucks maintained in range grazing (T1). The Hb concentration and RBC count ranged from 8.16 to 9.87 g/dL and from 8.52 × 10^6^ to 12.45 × 10^6^/µL, respectively, which are within the reference values of 8–12 g/dL Hb and 8–18 × 10^6^/µL RBC for healthy goats (Jackson and Cockcroft 2002). However, the RBC count found in this study was higher than that reported in a recent study (0.47‒0.82 × 10^6^/µL) for goats fed water hyacinth (Fanta et al. 2024a). Nevertheless, it is lower than earlier reports of 17.58‒17.85 × 10^6^/µL for growing goats in Abu Dhabi (Zaher et al. 2022). The RBC levels in this study are comparable with values of 7.38‒13.62 × 10^6^/µL for West African goats (Dupe et al. 2018). The PCV observed in this study ranged from 22.98%‒35.97%, which was also within the range of 22%‒38% reported for healthy goats (Jackson and Cockcroft 2002). The PCV of the bucks was higher than that reported previously (1.73%‒2.53%) for the same goat breed with different management system; it might be due to differences in diet types and feeding regime.

RBC indices, including MCV, MCH and MCHC, are metrics that are vital for diagnosing anaemia and provide details regarding average cell size, haemoglobin content and haemoglobin concentration, respectively (Jiménez‐Penago et al. 2021). These indices did not vary among the treatment groups. Furthermore, the MCV, MCH and MCHC values obtained in this study were within ranges of 16–25 fL, 5–8 pg and 30–36 g/dL, respectively, for healthy goats (Sirois 1995). The MCV value found in this study (20.93‒21.90 fL) was lower than the value of 40.08‒42.20 fL reported by Fanta et al. (2024a) but closer to the range value of 17.5‒25.5 fL obtained by Belewu et al. (2025) for Red Sokoto goats. The MCH value was in the range of 6.19‒7.03 pg, which is within the clinically normal range, suggesting that the animals were not suffering from reticulocytosis or conditions related to haemolysis (Enos and Moore 2022). MCHC is the most accurate of the RBC indices. It may increase in cases of haemolysis, while it may decrease in instances of reticulocytosis and iron deficiency anaemia (Polizopoulou 2010). The MCHC value in this study ranged from 30.78 to 32.65 g/dL, which is within the laboratory reference value of 30‒35 g/dL. Therefore, the animals were free from risk of haemolysis and reticulocytosis.

RDW‐CV and RDW‐SD are estimates derived from red cell distribution curves and are carried out to gauge the RBC size variability in circulation (Moreira et al. 2025). RDW‐CV values in this study range from 11.70 to 12.27%; this value is closer to the values of 13.84‒14.37% reported for Woyto‐Guji goats (Fanta et al. 2024a) but lower than 23%‒35% for Norwegian goats (Reiten et al. 2015) and 22.25%‒35.25% for Damascus bucks (Mohammed et al. 2016). Higher RDW concentrations indicate several conditions, including anaemia, sickle cell disease, autoimmune or myelodysplastic syndrome and deficiencies in vitamin B12 and folic acid (Jiménez‐Penago et al. 2021). WBCs play a prominent role in disease resistance, especially with respect to the generation of antibodies (Ahmad et al. 2022). The WBC count in this study ranged from 13.65 to 16.16 × 10^3^/µL. This range is comparable to the WBC values of 13.68–14.38 × 10^3^/µL reported for growing goats in Abu Dhabi (Zaher et al. 2022). Compared with the laboratory reference value of 4–13 × 10^3^/µL (Sirois 1995; Jackson and Cockcroft 2002), the value obtained in this study is far greater. The higher WBC count in the bucks suggests that the animals had an active immunological reaction against toxicity and contamination.

### 4.2 Serum Biomarkers

Serum biochemical markers are used to monitor and assess nutritional and physiological conditions effectively, as well as the variability of environmental challenges (Perera et al. 2022). The serum protein for the study animals ranged between 6.22 and 8.95 g/dL. These values are slightly greater than the normal range of 6.0 to 7.00 g/dL for healthy goats (Malecky et al. 2017). According to Mohammed et al. (2016), goats with a total protein value below 4.2 g/dL exhibit rumen compaction; hence, this phenomenon was not the case in the current study. Wang et al. (2023) reported that a higher serum protein is an indication of high intake of concentrates, particularly grains. The lower serum protein level in T1 (range grazing bucks) might be due to the fact that bucks in this group did not consume concentrate diets. Serum protein levels generally improved with the concentrate ratio, suggesting that a greater amount of crude protein (amino acids) was consumed from the concentrate diets, as was also reported by Soul et al. (2019). It also suggests that hepatic protein synthesis increased with the concentrate ratio. Consequently, the lower serum protein level in the range grazing group may suggest a protein deficiency in the range forages.

The serum albumin content for all treatments was within the healthy range of 2.70‒3.90 g/dL (Jackson and Cockcroft 2002). Zhou et al. (2026) indicated that low serum albumin levels are suggestive of liver dysfunction; nonetheless, other indicators of liver function, AST, ALT and AP, were similar among treatments, indicating diets did not negatively influence the liver. The AST, ALT and AP concentrations in this study fell within clinically healthy ranges of 0‒300, 15.3‒52.31 and 66‒230 μ/L, respectively (Jackson and Cockcroft 2002). Therefore, this study suggests that bucks in neither production system suffered from liver impairment. The serum urea levels (5.81‒6.44 mmol/L) in the current study are slightly higher than those reported by Soul et al. (2019) 4.87‒5.62 mmol/L. Nevertheless, they fell within the range of 3.6 to 9.2 mmol/L reported by Kaneko et al. (2008) for healthy animals. The current results support the reports of Fanta et al. (2024b), which indicated that dietary treatment had no impact on serum urea concentrations. Creatinine concentration in the present study is also within an acceptable range of 0.6‒1.06 mg/dL (Jackson and Cockcroft 2002) for all treatments.

The blood glucose concentration was elevated in concentrate‐fed bucks compared to the range‐grazing animals. The enhanced nutrient availability from concentrate diets might have contributed to this elevation at T2, T3 and T4. The glucose level recorded in this study (43.50‒56.25 mg/dL) is lower than the recent finding of 69.75‒84.50 mg/dL for the same breed of goat fed water hyacinth (Fanta et al. 2024b). The glucose level in T1 animals was also below the normal range for healthy goats (50‒75 mg/dL) (Jackson and Cockcroft 2002). Possible factors contributing to lower blood glucose in this group (T1) may include physical activity during field grazing and environmental stress. Glucose levels that fall below the normal range indicate hypoglycaemia, whereas elevated levels indicate hyperglycaemia (Habibu et al. 2021). The animals in T1, therefore, suffered from hypoglycaemia.

The present study also revealed that the serum testosterone concentration in the concentrated‐based production systems was greater than that in the range grazing bucks. This finding aligns with the study conducted by Al‐Yahyaey et al. (2023), which described that supplementation with *Spirulina platensis* led to increased blood testosterone levels in Sahrawi and Jabbali goat bucks. This finding is also in agreement with a previous investigation by Delgadillo et al. (2021), who reported increased testosterone levels with nutritional intervention. The micronutrients obtained through dietary sources play crucial regulatory roles in testosterone biosynthesis and secretion (Lin et al. 2022). Liu et al. (2025) noted that diet influences the composition and abundance of gut microbes, which in turn affects circulating testosterone levels. The elevated testosterone levels in concentrate‐based production systems (T2, T3 and T4) may be due to the increased availability of nutrients from the concentrates. Therefore, increased testosterone in these groups (T2, T3 and T4) is commonly associated with improved libido, spermatogenic activity and overall male fertility potential, so these findings imply that concentrate feeding may enhance reproductive performance in bucks through endocrine modulation.

### 4.3 Serum Minerals

Macro minerals are essential for livestock growth, health and reproduction and are regarded as critical nutrients since their deficiency can hinder the utilization of other nutrients and reduce overall performance (Mayasula et al. 2021). Greater serum calcium in T4 (compared to T1) indicates that this ration supplied more bioavailable Ca or improved intestinal absorption, which is important for neuromuscular function, bone health and reproductive events such as sperm maturation. This result agrees with the findings of Giger‐Reverdin et al. (2014), who observed increased serum Ca in dairy goats due to concentrate feeding. Such improvements prevent hypocalcaemia‐related issues such as reduced fertility, which is common in ruminants on basal diets low in Ca bioavailability. All serum minerals except Cl fell within the recommended ranges for healthy goats (e.g., K 3.6–5.6 mmol/L, Na 140–154 mmol/L, P 1.5–2.5 mmol/L, Ca 2.2–2.8 mmol/L) (MSD Veterinary Manual 2024).

### 4.4 Correlational Studies

The Pearson correlation coefficient indicated a range of associations, from positive to negative associations, and from low to strong, among the haematological and serum metabolites. RBC count was strongly correlated with PCV, indicating that increased RBC count drives increased PCV. This result agrees with the findings of Fanta et al. (2024a), who reported a significant positive correlation between RBC and PCV (*r* = 0.76) for Woyto‐Guji goat breeds. RBC also exhibited a strong positive correlation with Hb, which is in line with the observations of Soca et al. (2025), who found a strong positive association between the parameters (*r* = 0.82) in Creole goats on the southern coast of Peru. Corresponding to the present study, the same authors (Soca et al. 2025) also reported a weak correlation for PCV versus MCH (*r* = −0.25), RBC versus MCH (*r* = −0.34) and RBC versus MCHC (*r* = −0.11). This result also supports the findings of Karki et al. (2021), who stated that RBC indices (MCV, MCH and MCHC) in pasture‐raised goats were negatively related to RBC. The near‐perfect correlation between RDW–CV and RDW–SD in this study is consistent with the findings of Karki et al. (2021), who found a strong positive connection between the traits (*r* = 0.80).

Serum protein was strongly correlated with albumin, which aligns with the results reported by Fanta et al. (2024b) for Woyto‐Guji goats. This finding also agrees with the results of Djuricic et al. (2011), which indicated a strong positive correlation between serum protein and albumin in Boer and Saanen goats. The albumin‒globulin ratio is strongly correlated with serum albumin concentration; however, it was negatively correlated with the globulin concentration. This implies that a higher albumin‒globulin ratio corresponds to more albumin in the blood, indicating that the goat bucks have healthy liver function. Globulin was weakly correlated with albumin, which is in agreement with a previous report by Fanta et al. (2024b) in the same breed of goats. Testosterone showed a strong positive correlation with glucose, emphasizing the connection between energy availability and the level of reproductive hormones in the animal.

## 5 Conclusions

Haematological parameters, including haemoglobin concentration, red blood cell count and packed cell volume, were increased in bucks fed higher concentrate diets in T3 and T4 than T1. Serum total protein, albumin, glucose and testosterone concentrations were also significantly higher in the concentrate‐fed bucks (T2‒T4) than in those managed under range grazing (T1). In addition, the calcium concentration of the animals in T3 and T4 was significantly elevated compared to those in T1. However, the feeding systems (range grazing vs. indoor concentrate‐based) had no considerable effect on the mean corpuscular volume, mean corpuscular haemoglobin, mean corpuscular haemoglobin concentration, white blood cell count, red cell distribution coefficient of variation and red cell distribution standard deviation values. The serum biochemistry profiles, including globulin, albumin‐to‐globulin ratio, AST, ALT, AP, urea, creatinine, potassium, sodium, chloride and phosphorus, also remained unaffected. Although some parameters, such as white blood cell counts and serum chloride levels, deviated from the reference ranges for healthy goats, none of the bucks showed an inflammatory response or toxicity.

## Author Contributions

**Selamu Abraham**: conceptualization, investigation, writing – original draft, methodology, software, data curation, formal analysis. **Yisehak Kechero**: conceptualization, methodology, supervision, writing – review and editing, visualization, validation, resources. **Jane Wamatu**: conceptualization, validation, supervision, resources, funding acquisition, project administration.

## Funding

This work was supported by Arba Minch University and the International Centre for Agricultural Research in the Dry Areas (ICARDA). The funders provided financial support for fieldwork but had no role in the decision to publish or in manuscript preparation.

## Ethics Statement

The authors confirm that the ethical policies of the journal, as noted on the journal's author guidelines page, have been adhered to and the appropriate ethical review committee approval has been received. The study protocol was reviewed and approved by the ethics committee of Arba Minch University Institutional Review Board (IRB), under reference number RCO/CAS/04/2024.

## Conflicts of Interest

The authors declare no conflicts of interest.

## Data Availability

The data that support this study will be made available from the corresponding author upon reasonable request.
